# A Multi-Modal Analysis of the Freezing of Gait Phenomenon in Parkinson’s Disease

**DOI:** 10.3390/s22072613

**Published:** 2022-03-29

**Authors:** Luca Mesin, Paola Porcu, Debora Russu, Gabriele Farina, Luigi Borzì, Wei Zhang, Yuzhu Guo, Gabriella Olmo

**Affiliations:** 1 Department of Electronics and Telecommunications, Politecnico di Torino, Corso Duca degli Abruzzi 24, 10129 Torino, Italy; luca.mesin@polito.it (L.M.); paola.porcu@studenti.polito.it (P.P.); debora.russu@studenti.polito.it (D.R.); 2 Neurology Unit, Azienda Ospedaliera Universitaria di Sassari, Viale San Pietro 10, 07100 Sassari, Italy; gabriele.farina@gmail.com; 3 Department of Control and Computer Engineering, Politecnico di Torino, Corso Duca degli Abruzzi 24, 10129 Torino, Italy; luigi.borzi@polito.it; 4 Department of Neurology, Neurobiology and Geriatrics, Beijing Institute of Geriatrics, Xuanwu Hospital of Capital Medical University, Beijing 100053, China; wzhang_pd@hotmail.com; 5 Department of Neurology, The Affiliated Hospital of Xuzhou Medical University, Xuzhou 221006, China; 6 School of Automation Science and Electrical Engineering, Beihang University, Beijing 100191, China; yuzhuguo@buaa.edu.cn

**Keywords:** Parkinson’s disease, Freezing of Gait, multi-modal analysis, inertial sensors, electroencephalogram (EEG), skin conductance (SC), machine learning, support vector machine (SVM), k-nearest neighbor (kNN)

## Abstract

Background: Freezing of Gait (FOG) is one of the most disabling motor complications of Parkinson’s disease, and consists of an episodic inability to move forward, despite the intention to walk. FOG increases the risk of falls and reduces the quality of life of patients and their caregivers. The phenomenon is difficult to appreciate during outpatients visits; hence, its automatic recognition is of great clinical importance. Many types of sensors and different locations on the body have been proposed. However, the advantages of a multi-sensor configuration with respect to a single-sensor one are not clear, whereas this latter would be advisable for use in a non-supervised environment. Methods: In this study, we used a multi-modal dataset and machine learning algorithms to perform different classifications between FOG and non-FOG periods. Moreover, we explored the relevance of features in the time and frequency domains extracted from inertial sensors, electroencephalogram and skin conductance. We developed both a subject-independent and a subject-dependent algorithm, considering different sensor subsets. Results: The subject-independent and subject-dependent algorithms yielded accuracies of 85% and 88% in the leave-one-subject-out and leave-one-task-out test, respectively. Results suggest that the inertial sensors positioned on the lower limb are generally the most significant in recognizing FOG. Moreover, the performance impairment experienced when using a single tibial accelerometer instead of the optimal multi-modal configuration is limited to 2–3%. Conclusions: The achieved results disclose the possibility of getting a good FOG recognition using a minimally invasive set-up made of a single inertial sensor. This is very significant in the perspective of implementing a long-term monitoring of patients in their homes, during activities of daily living.

## 1. Introduction

Parkinson’s disease (PD) is a neurodegenerative condition caused by the selective degradation of dopaminergic neurons, especially in the mesencephalic *substantia nigra pars compacta*, which is involved in several processes related to movement and cognition [1,2]. Idiopathic PD is considered a multi-factorial pathology, due to the complex interaction between multiple genetic and environmental risk factors. The four *cardinal signs* of PD are tremor at rest, rigidity, bradykinesia and postural instability [3]. Non-motor symptoms encompass depression, autonomic dysfunctions and, in the advanced stages of the disease, possible cognitive impairment and dementia [4].

At present, a curative therapy of PD does not exist; however, both pharmacological and surgical treatments can effectively manage symptoms for several years. Levodopa is still the gold standard for the control of PD motor symptoms, even though it can induce involuntary movements (i.e., dyskinesia) and response fluctuations (wearing off, OFF periods), especially after several years of administration [5]. The evolution of PD is monitored with the help of internationally validated scales and questionnaires. The MDS-UPDRS (Unified Parkinson’s Disease Rating Scale, promoted by the Movement Disorder Society - MDS) is widely used to assess the severity and progression of the disease [6].

Freezing of Gait (FOG) is one of the most troublesome and enigmatic motor complications of PD. It is defined as a *brief, episodic absence or marked reduction of forward progression of the feet despite having intention to walk* [7]. Its occurrence is related to high risk of falls, reduced functional independence and impaired quality of life [8]. Recent molecular imaging studies have suggested a causal role of the supraspinal locomotor network involving the primary motor cortex, the supplementary motor area, the parietal cortex, the basal ganglia, the subthalamic nucleus, the mesencephalic locomotor region and the cerebellum [9]. However, the pathogenesis of FOG is not completely known and possibly not unique. Moreover, several sub-types of FOG are recognized and different classification criteria co-exist [10,11].

The clinical evaluation of FOG is difficult and highly subjective. In fact, given their sporadic nature, FOG episodes seldom occur during the brief medical examination, as their incidence is dependent on the pharmacological status and on the patient’s attention devoted to gait. Moreover, a moderate emotional stress (e.g., a medical visit) may inhibit FOG [12]. Consequently, at present, the FOG assessment is mainly based on diaries and questionnaires self-managed by the patients themselves [13] and whose reliability is questionable. Direct observation of the phenomenon may be improved using elicitation strategies, e.g., proper walking and dual tasks administered to the patients [13,14,15]. However, this approach is time-consuming and not compatible with the everyday clinical practice. Thus, it is clear that FOG evaluation can greatly benefit from objective data, collected continuously with proper sensors placed on the patient’s body and worn during activities of daily living (ADLs) [16]. In perspective, this could disclose the possibility of performing the evaluation in the patient’s own home, so enabling a more objective assessment of motor fluctuations and complications during ADLs. Another challenge is the possibility to identify (or predict) FOG events in due time so as to help their resolution (or even prevent their occurrence) with the aid of visual, auditory, or somato-sensorial stimuli [17,18].

In the literature, the following sensors have been addressed in the study of the FOG phenomenon.

- Electromyography (EMG) measured on lower limbs [19]. Typical patterns have been detected in muscle activity preceding and following FOG episodes [20,21].
- Electroencephalography (EEG) data were used to detect and predict FOG occurrence [22]. Coherence with EMG close and during FOG episodes has been revealed [23], along with bilateral cortical excessive synchronization during locomotion [23], an increase in the theta-band power in frontal and central areas [22,24], a decrease in power during the voluntary arrest compared to FOG [25] and a less complex cortical activity during the transition periods [26].
- Skin conductance (SC), encompassing selective information useful for FOG prediction and detection [27], even considering the heavy subject-dependency.
- Inertial sensors. Single or multiple sensors have been placed on several body segments (e.g., legs [28], wrist [29], waist [30]) for FOG detection [29,31] and prediction [28,29,32]. In particular, accelerometers are widely employed, due to their low energy consumption and cost (in particular, those embedded in smartphones [15,33]). Significant information is provided by the *Freeze Index*, defined as the ratio between the power contained in the so-called *freeze band* 3–8 Hz [34] and that in the locomotion band 0.5–3 Hz [29,35]. Entropy and statistical parameters such as mean value, standard deviation and variance are other sensible metrics [28,31]. Several features, extracted from both the time and frequency domain and different machine learning (ML) algorithms have been employed to classify FOG and pre-FOG events [36].

The integration of information from multiple sensors, considering a multi-modal acquisition and processing system, could be useful to improve the identification of FOG. For example, information from several sensor types was employed to improve the distinction between complete akinesia and the standing or trembling FOG sub-type, which represents a very difficult task using inertial sensors in a non-supervised environment [15].

Information from EEG and inertial sensors on lower limbs were combined in [35]. The comparison of the multi-modal and single-model (inertial sensors only) showed that EEG was capable of improving the specificity but not the sensitivity of the system in the detection of FOG. Moreover, impaired results were obtained (i.e., sensitivity 62%, specificity 62%), possibly due to the small feature set considered. A multi-modal analysis combining information from EEG, electrocardiogram (ECG), electro-oculogram (EOC), accelerometers and foot-switches was proposed in [25]. Merging features extracted from each sensor resulted in very good performance (i.e., sensitivity 97%, specificity 96%). However, statistical analysis did not identified any significant difference between the multi-modal approach and the accelerometers-only based approach. A FOG prediction system based on pressure sensors positioned on insoles was discussed in a recent paper [37]. Finally, the ECG was analyzed together with SC, observing significant variations just before the occurrence of a FOG episode [27].

A multi-modal analysis, which is likely more effective in evaluating the FOG phenomenon, has the obvious drawback of being more invasive, less comfortable, more expensive, power consuming and hardly feasible in the non-supervised home environment. The objective of the present study is to evaluate the possible improvements in FOG detection using several sensors, compared with simpler configurations (e.g., a single inertial measure). Our goal is to understand which signals are the most significant to detect/predict FOG and whether they carry complementary information. This could help choosing the best trade-off between performance and complexity, defining the simplest possible system able to satisfactorily monitor FOG during ADL.

Unlike previous studies [25,35], the walking tasks used in this study were defined to resemble those of daily living. Moreover, as a reduced feature set may result in poor performance [35], a very large feature set was extracted from each sensor in the present work. This was done in order to take full advantage of the prediction capability of each sensors’ configuration.

## 2. Materials and Methods

In this section, we describe the data and processing steps performed to detect FOG episodes. Specifically, in Section 2.1 we describe the dataset used for the analysis, including information regarding the population enrolled, the sensor systems used and the experimental procedures. In Section 2.2, we describe the data processing steps, including pre-processing (Section 2.2.1 and Section 2.2.2), feature extraction (Section 2.2.3) and the implementation of both the subject-independent (Section 2.2.4) and subject-dependent (Section 2.2.5) classification algorithms.

### 2.1. Dataset

The dataset includes recordings from 12 patients with PD, collected in Beijing Xuanwu Hospital, China [38]. The inclusion criteria were:

- PD patients subject to FOG during OFF periods;
- PD patients able to walk independently during OFF periods;
- No severe vision or hearing impairment;
- No sign of dementia or other neurological/orthopedic disease.

**Figure 1:**
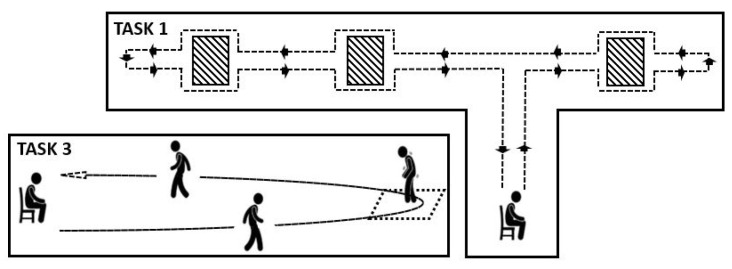
Sketch of the walking tasks executed during experimental procedures.

The research was conducted according to the declaration of Helsinki and ethical approval was obtained from the Ethics Committee of Xuanwu Hospital, Capital Medical University, Beijing, China (No. 2019-014). Written informed consent was obtained from all participants. EEG, EMG, ECG, EOG, SC, inertial data from sensors positioned on the lateral tibia of the left and right legs, fifth lumbar spine and wrist were recorded during the experiments. EEG and EMG were acquired using a 32-channel wireless MOVE system (BRAIN PRODUCTS, Germany), while inertial signals and SC were acquired using self-designed hardware subsystems based on a TDK MPU6050 6-DoF accelerometer and gyroscope, with a STMicroelectronics STM32 processor. The experimental procedure was designed to include FOG triggering tasks. After reading and signing informed consent, participants were subject to a physical examination. They did not take any drugs within 2 h before the experiment to ensure that they were in OFF conditions. After wearing the multi-modal sensory equipment, they were asked to complete the following walking tasks, schematically represented in Figure 1.

**Task 1-**  When ready, the participants had to rise from a chair and walk up to a narrow space between the room and a corridor. Then, they turned right and walked into the corridor. After bypassing a first obstacle (e.g., a chair), they went straight along the narrow corridor, made a U-turn at the end of the corridor and went along in the opposite direction. They had to bypass three further obstacles, reach the left end of the corridor, make another U-turn, bypass two obstacles, reach the door of the room, enter the room, walk back to the chair and sit down.
**Task 2-**  Consisted of a repetition of Task 1.
**Task 3-**  Patients were asked to perform a turn in a limited space and a square was drawn on the ground for this aim. When the patient was ready, they had to stand up from the chair, walk to the square mark, make a U-turn in the narrow square region and then walk straight back to the chair and sit down.
**Task 4-**  Consisted of a repetition of Task 3.

A video was recorded during the whole experiment for the physicians to define the start/end of each FOG episode. In this study, we included inertial signals from the left tibial and wrist positions, EEG and SC data, with the aim of seeing which sensors could be excluded while ensuring a marginal reduction in performance. Other inertial sensors, ECG and EMG were excluded as they were not available for all subjects or they did not have sufficient quality. Even recognizing the importance of these additional data (e.g., the use of both inertial and EMG allowed obtaining outstanding FOG detection accuracy in [21]), excluding them has the advantage of making the recording system economic, simple and energy saving. Moreover, even though we recognize that ECG can be closely related with the physiological changes during FOG and can be useful for predicting FOG, the collection of such data can be challenging [27], especially if performed in non-supervised conditions.

For the sake of consistency, all signals were re-sampled at a frequency of 500 Hz. The information on the employed sensors is reported in Table 1 and Table 2. Patients’ main demographic and clinical characteristics are summarized in Table 3.

### 2.2. Data Processing

**Figure 2:**
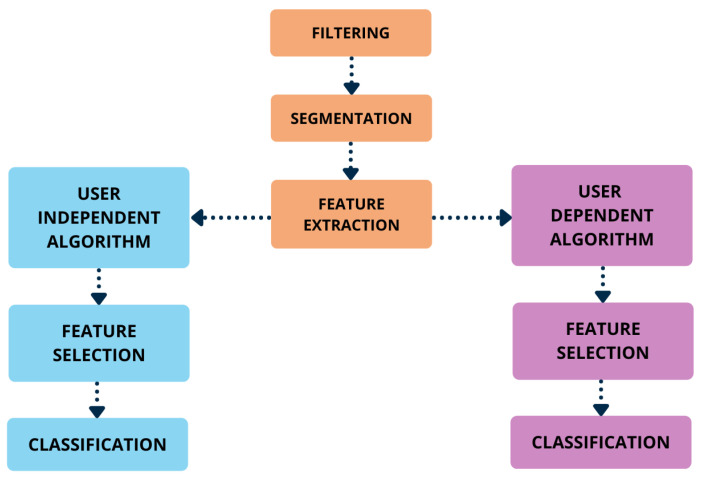
Diagram of the implemented FOG detection algorithms.

The diagram shown in Figure 2 represents an overview of the algorithms implemented in this study. After proper filtering, signals were segmented and several features were extracted in each window, as described later on in this section.

As mentioned, two different algorithms were implemented, namely the subject-independent algorithm (SIA) and the subject-dependent algorithm (SDA). As for the SIA, feature selection (FS) was performed on the entire dataset, encompassing data from all involved subjects. On the other hand, in SDA, single patient data were employed. The reduced feature sets output by the FS procedure in either case were used to train, validate and test different classifiers and the achieved classification performances were compared. Each step of the two implemented algorithms is described in detail in Section 2.2.4 and Section 2.2.5.

#### 2.2.1. Filtering and Standardization

First of all, signals (here indicated by *s*) were normalized using the z-score normalization formula reported in Equation (1), in order to reduce inter-patient variability

(1) $$s^{\prime }=\frac{s-mean(s)}{std(s)}$$

Then, data were processed as follows.

**Inertial signals**: acceleration and angular velocity signals were band-pass filtered in the band 0.5–16 Hz using 4th order high-pass and 5th order low-pass Butterworth filters. Notice that during walking the acceleration spectrum mainly lies in 0.5–3 Hz (locomotion band), while during FOG it is centered in 3–8 Hz (freeze band).

**EEG**: a longitudinal bipolar configuration was used to reduce the common mode and obtain more selective information, resulting in a total of 18 channels. Then, the signals were band-pass filtered in the band 1.6–30 Hz using 5th order high-pass and 6th order low-pass Chebyshev filters. The blinking artifacts and the EMG artifacts were removed using the toolbox proposed in [39].

**SC**: the signal was pre-processed, as in [27]. Specifically, the galvanic resistance provided by the sensor was converted into skin conductance by performing the reciprocal of each resistance value. As the signal exhibits slow variations over time, high-frequency components and artifact transients are not significant and must be suppressed. To this end, a 5th order Butterworth low-pass filter with a cutoff frequency of 2 Hz was applied, while the rapid transients were removed using a moving average of 3–6 s centered on the artifact. Subsequently, a 5th order Butterworth high-pass filter with cutoff frequency of 0.5 Hz was applied to extract the phasic component (skin conductance response-*SCR*); the tonic component (skin conductance level-*SCL*) was then obtained by direct subtraction [40]:

(2) SCL=SC−SCR

#### 2.2.2. Labeling and Segmentation

**Figure 3:**
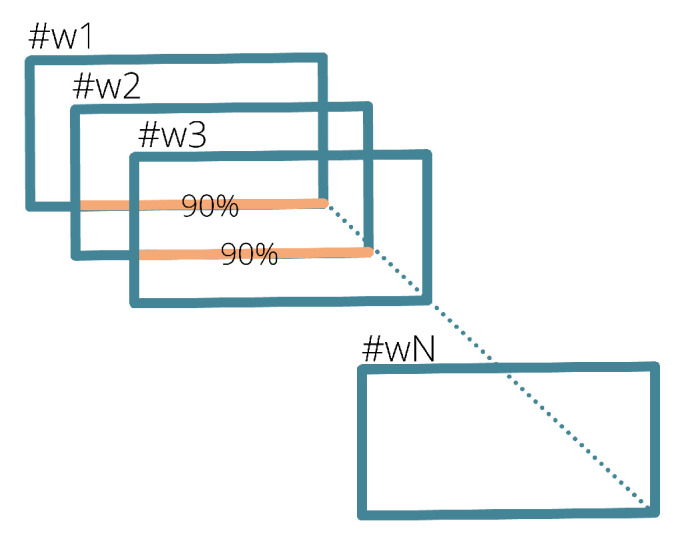
Signal segmentation scheme with 90% overlapping windows.

The labeling of FOG episodes was performed by specialized clinicians via direct inspection of video recordings. Signals were divided into windows (w) of fixed duration, properly overlapped to avoid loss of information (Figure 3). The window length is inversely proportional to the temporal resolution of the detection algorithm [15]; for this reason, a short window duration allows the identification of short FOG episodes. However, choosing too short of windows heavily affects computation efficiency. In order to achieve a trade-off between these aspects, 3 s windows with 90% overlap were selected. Each window was labeled as “non-FOG” or “FOG” based on the classification of the largest number of samples.

#### 2.2.3. Feature Extraction

Many time- and frequency-domain features were computed, as suggested in the literature on FOG prediction and detection [15,22,25,26,27,28,29,30,31,32,34,35]. The features extracted from the inertial signals measured at the leg were calculated on the three components independently (i.e., vertical, medial-lateral and antero-posterior). On the other hand, at the wrist level, features were computed from the signal norm, besides the three components separately, according to [32]. Regarding the EEG, the features were extracted from all the bipolar channels. Moreover, the magnitude squared coherence (MSC) was computed for each pair of channels in the different frequency bands (i.e., delta, theta, alpha, beta1 and beta2). Concerning the skin conductance, the phasic component, its first and second derivatives and the tonic component were used. A separated feature set was obtained for each sensor and normalized using the range normalization formula reported in Equation (3).

(3) $$f^{\prime }=\frac{f-min(f)}{max(f)-min(f)}$$

Table 4 reports the full list of features extracted in this work, grouped by sensor type and domain, i.e., time or frequency. The total number of features extracted from each sensor, considering all signal components, is reported in Table 5.

#### 2.2.4. Subject-Independent Algorithm

This algorithm carries out the feature selection from data related to all patients, thus identifying the most informative characteristics independently of the subject. In particular, features with at least moderate correlation with the output (r ≥ 0.35) and with *p*-value < 0.05 were retained and redundant features (i.e., with cross-correlation r ≥ 0.86) suppressed, keeping only those with maximal correlation with the output.

The selected features were input to two ML models, namely k-nearest neighbor (kNN) and support vector machine (SVM). The main advantage of kNN lies in its simple implementation and the ability to identify nonlinear decision boundaries; on the other hand, SVM is a typical choice in FOG detection studies as it generally yields high performance [15,28]. Leave-one-subject-out (LOSO) was performed for both the validation and the test procedure. LOSO consists of training the model with data from all patients except one, which is used for testing. This provides a robust and realistic assessment of the classifier performance. For each patient under test, models were optimized using data from the remaining N-1 patients. Specifically, during the validation phase, the model parameters were optimized using LOSO validation. The entire procedure is reported in Algorithm 1, where *parameterList* refers to the list of parameters reported in Table 6.
**Algorithm 1** Algorithm for model optimization and performance evaluation in the Subject-Independent case.**procedure**optimizedModel(Data), performance(Data)                       ▹    **for** i←1 to *N* **do**                        ▹ Perform N times test procedure        [trainingSet]← data from all subject except for $i^{th}$                      ▹        [testSet]← data from $i^{th}$ subject                                ▹        **for** j←1 to N−1 **do**                   ▹ Perform N-1 times validation procedure           [trainingSet]← data from trainingSet except for $j^{th}$ subject                         ▹           [validationSet]← data from $j^{th}$ subject of trainingSet                        ▹           **for** each model ← [SVM,k-NN] **do**                                   ▹               **for** each parameter ← [parameterList] **do**                            ▹                   trainedModel ← Train(model(trainingSet,parameters))                   prediction← predict(trainedModel(validationSet,parameters))                   performanceVal← performance(prediction,label)               **end for**           **end for**           bestPerformance← max(performance)           optimalParameters← parameters(bestPerformance)                        ▹        **end for**        prediction←predict(model,optimalParameters,testSet)                      ▹        performanceTest←performance(prediction,label)                     ▹    **end for**    **return** model,optimalParameters,performance                          ▹**end procedure**

The choice of the optimal model was made by evaluating both accuracy and F-score. Specifically, the model that exhibited the maximum value for both metrics was selected; if the same model did not yield the maximum value for both metrics, that providing the maximum F-score was chosen. Once the model was selected, the classification performance was evaluated by means of accuracy, precision, sensitivity, specificity and F-score on the test set. The definitions of the metrics are reported in Equations (4) and (5).

(4) $$Sensitivity=\frac{TP}{TP+FN}\;Specificity=\frac{TN}{TN+FP}\;Precision=\frac{TP}{TP+FP}$$

(5) $$Accuracy=\frac{TP+TN}{FP+FN+TP+TN}\;F-score=\frac{2\;\cdot \;Precision\;\cdot \;Sensitivity}{Precision+Sensitivity}$$

*TP* (*TN*), *FP* (*FN*) being the number of true positive (negative) and false positive (negative) classifications. *TP* is defined as the number of those windows labeled as FOG and classified as FOG by the algorithm; *FP* refers to non-FOG data identified by the model as FOG; *FN* represents the number of undetected windows labeled as FOG; finally, *TN* is defined as the number of non-FOG windows correctly identified by the classification algorithm.

#### 2.2.5. Subject-Dependent Algorithm

This algorithm aims to select significant features of each subject by carrying out the feature selection on the data related to each individual patient. The feature set dimension was reduced by keeping only features with a Pearson correlation coefficient r ≥ 0.4 with the target and with a corresponding *p*-value < 0.05; subsequently, the redundant features were suppressed (cross-correlation r ≥ 0.86). The correlation coefficient threshold was selected according to Evans’ classification [41], as a Pearson’s correlation coefficient value of 0.4 represents the edge between weak and moderate correlation.

The reduced feature sets were used to train, validate and test the ML models. A 10-fold cross-validation was employed for validation and model optimization, while a leave-one-task-out (LOTO) has been used for the test phase. This choice was made considering that, for some of the involved subjects, FOG episodes were observed during the execution of only two activities; therefore, in these cases, LOTO cannot be used for both steps.

The validation phase has been carried out for the ML models by tuning the same hyper-parameters previously discussed. The selected model was used to evaluate the classification performance through the same metrics previously addressed, reported in Equations (4) and (5).

*Majority voting* (MV) was applied both for SIA and SDA. It removes localized classification errors considering more subsequent windows and selecting the most represented activities within them. Five epochs have been used in this study, corresponding to approximately 4 s (considering windows of 3 s with 90% overlap), thus keeping a time resolution compatible with short FOG episodes.

## 3. Results and Discussion

Among the 18 recruited patients, we completed 12 valid experiments and made available 3.7 h of data (17 ± 10 min each patient). The number of detected FOG events was 334 (25.7 ± 17 for each patient), with a total FOG duration of 88.3 min (6.8 ± 7.6 min for each patient). The duration of each FOG event ranged from 1 to 201 s; approximately 35% of episodes lasted less than 5 s and about 50% of episodes lasted less than 10 s, consistently with the clinical evidence. FOG episodes lasting less than 3 s were excluded from the analysis, as inferior to the time resolution after MV; this led to a total number of 264 events considered. The rest of this section reports the classification results achieved by the SIA (Section 3.1) and the SDA (Section 3.2).

### 3.1. Subject-Independent Algorithm

Table 7 lists the features selected when using data from all patients. The FS procedure extracted 12 (11) features from the acceleration (angular velocity) signal and all of them were obtained from the sensor positioned on the leg. During FOG, a shift toward the high frequencies was observed, as demonstrated by the increase in median and dominant frequency, freeze index and freeze ratio and a decrease in locomotion band power. Moreover, a lower movement intensity was observed, as demonstrated by the decreasing of RMS value. Finally, movement regularity decreased, as proven by the increase in the spectral entropy.

**Figure 4:**
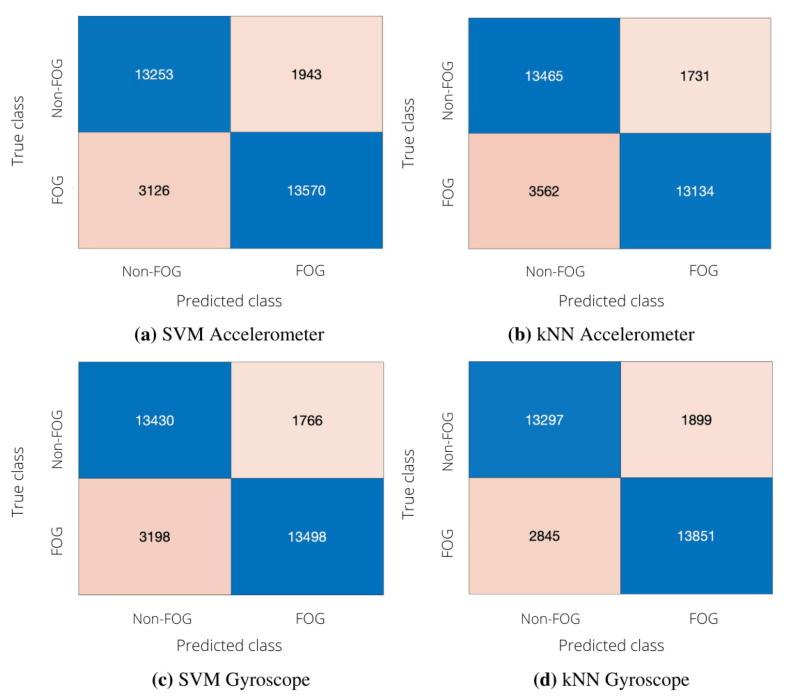
Performance of the subject-independent algorithm in uni-modal configuration. (**a**) Confusion matrix obtained with SVM; left lateral tibial accelerometer. (**b**) Confusion matrix obtained with kNN; left lateral tibial accelerometer. (**c**) Confusion matrix obtained with SVM; left lateral tibial gyroscope. (**d**) Confusion matrix obtained with kNN; left lateral tibial gyroscope.

In order to investigate the effect of reducing the number of sensors, we compared uni-modal classifications (performed using either the accelerometer or the gyroscope data) and a multi-modal classification involving both signals. The confusion matrices of uni-modal classifiers are depicted in Figure 4, whereas Table 8 reports the performance of uni-modal classification in terms of sensitivity, specificity, accuracy, precision and F-score.

From Table 8a, it can be seen that SVM and kNN provided similar performance when considering acceleration signals. In more detail, kNN provided fewer FP and more FN than SVM, thus leading to larger specificity and lower sensitivity with respect to SVM. As for the results achieved using the gyroscope, it is possible to notice that both SVM and kNN provide classification metrics comparable to those achieved with the accelerometer (Table 8b). For example, the F-score values obtained by the two uni-modal classifiers are similar in the case of SVM and differ by 2% in the case of kNN.

**Figure 5:**
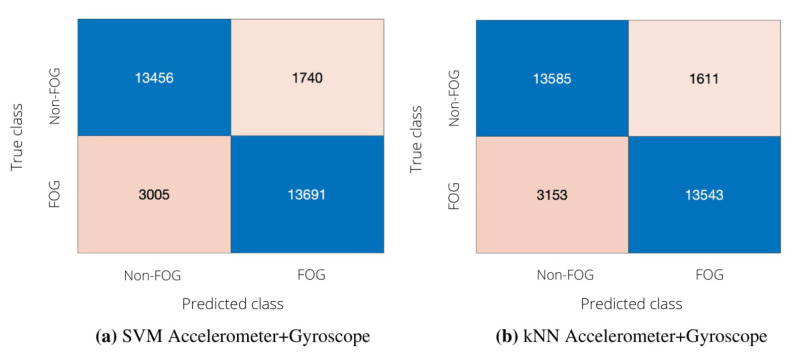
Performance of subject-independent algorithm with multi-modal classification, using left lateral tibial accelerometer and gyroscope. (**a**) Confusion Matrix obtained with SVM. (**b**) Confusion Matrix obtained with kNN.

The performance of the multi-modal classification, which combines accelerometer and gyroscope data, is reported in Figure 5 in terms of confusion matrices and in Table 9 in terms of accuracy, precision, specificity, sensitivity and F-score. Both SVM and kNN multi-modal classifications yield slightly improved performance with respect to the uni-modal classification employing the accelerometer alone; however, an improvement in F-score of 1% is not consistent, thus suggesting the choice of a simple and cost-effective solution using the accelerometer alone.

### 3.2. Subject-Dependent Algorithm

When considering data from each patient separately, the FS procedure selected significant features from the inertial sensors on the left lateral tibia and on the wrist, together with some EEG characteristics, while no feature from SC data was selected. In particular, the search for subject-dependent information led to significant correlations also for signals, which were not included in the subject-independent algorithm. As for the inertial signals recorded from the sensor on the tibia, during FOG a shift towards high frequencies was observed, with a consequent increase in the dominant and median frequencies and in freeze indices. Moreover, a decrease in the range of motion was observed by a decrease of RMS and maximum amplitude of the signal. Finally, the low number of peaks suggests a reduced forward progression. Concerning the inertial signals recorded at the wrist, an increase in the dominant and median frequencies in the vertical direction was observed during FOG. As for the other features, different patterns were observed among the patients, thus suggesting that movements of the upper limbs during FOG are rather heterogeneous across subjects.

The EEG signal was found to be informative in 5 out of 11 patients. All the selected features concern the frequency domain, especially for data recorded from the frontal-central and the central-parietal regions, which are likely involved in the phenomenon, as also reported in [22,24,26]. The features computed using the cross-spectra of channel pairs express, for some patients, a decrease in coherence in the delta band and in others an interesting increase in coherence in the beta1 and beta2 frequency bands. Although preliminary, this result may suggest an increase in the synchronization between central and parietal regions, in order to "by-pass" the most affected regions, usually localized in the central area.

In order to verify the relative importance of using multiple sensors, the following conditions were compared.

- Single-sensor classification, considering accelerometer data from the left tibial sensor only (*Minimal Setup*);
- Multi-sensor classification, using data from all sensors that provided significant features for each individual patients (*Complex Setup*), i.e., acceleration and angular velocity at tibial level, acceleration and angular velocity at wrist level and EEG.

**Figure 6:**
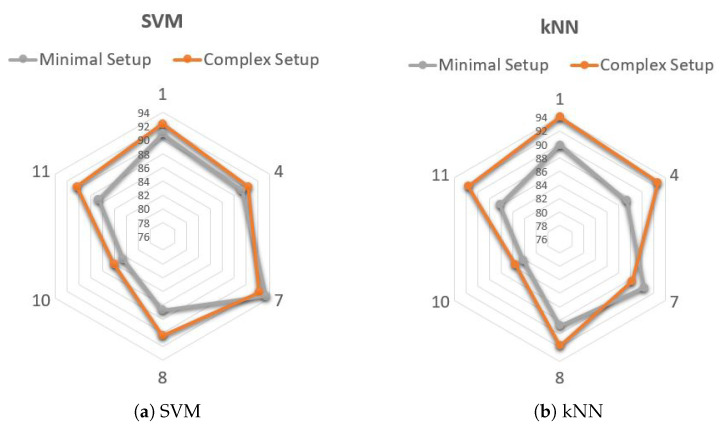
Subject-dependent algorithm. F-score (%) obtained with minimal (left tibial accelerometer alone) and complex (multi sensor) setup in each subject.

The F-scores achieved with the two configurations, using SVM and kNN classifiers, are reported in Figure 6. Only the F-scores of subjects for which relevant features were extracted from at least two sensors are reported; furthermore, in the case of Subject 10, EEG features were not selected. As can be seen, for both the SVM and kNN models, the F-score was similar between the two different configurations. In particular, in the case of SVM, the minimal and complex setups yielded F-scores in the ranges 82.6–93.4 and 84.1–92.3, respectively. The largest improvement provided by the complex setup was 3.75% (Subject 8). As for kNN, the minimal and complex setups yielded F-scores in the ranges 82.17–90.4 and 83.58–93.97, respectively, with the largest improvement of 5.34% observed in Subject 11. Even though these results are necessarily preliminary, due to the reduced number of subjects involved, the outcomes suggested that the complex setup provides quite limited performance improvements with respect to the minimal configuration. Hence, this latter may represent a good option in a context where low cost, low power consumption and patient’s comfort are key issues, as in the case of continuous monitoring during ADL. Table 10 shows the overall performance of SDA in terms of accuracy, sensitivity, precision, specificity and F-score. Only those patients for which the comparison between minimal and complex setup was possible (i.e., features selected from at least two sensors) are reported.

In order to validate the effectiveness of the minimal configuration, we verified the actual algorithm capability of detecting FOG episodes. Table 11 reports the number of real FOG episodes (as labeled by the clinicians after video inspection) and that provided by the subject-dependent and independent algorithms with minimal setup configuration. As evident from Table 11, the system was capable of detecting 232/264 FOG events (87.9%), with detection rate larger than 90% in all patients except for subjects 9 (who experienced FOG episodes during less than 4 s) and 11. These latter patients manifested several FOG episodes (22 and 36, respectively), with only a part of them recognized by the algorithm. This seems to prove that episodes of the same patient are not necessarily correlated.

## 4. Conclusions

In this study, we explored different types of sensors and positioning for FOG detection through optimized ML algorithms. A total of 12 patients with PD were included and 11 of them experienced FOG episodes while performing different motor tasks. We addressed both a subject-independent and a subject-dependent algorithm. In the former case, only inertial signals recorded from the left lateral tibia were selected, while in the latter case, features from EEG data and from the inertial sensor positioned on the wrist were also selected. The LOSO and LOTO validations yielded F-scores and accuracy in excess of 83%. As for SIA, the classification performance was compared considering either uni-modal (i.e., accelerometer and gyroscope alone) or multi-modal classification (sensors combination), obtaining similar results. As for SDA, a minimal configuration, including only the accelerometer positioned on the lateral left tibia, was compared with a more complex condition, including features selected from the other sensors (EEG and inertial sensors at wrist and leg). Although the use of multiple sensors results in a performance improvement, the difference was found to be limited. This suggests that the adoption of the minimal configuration could be a good compromise between classification accuracy and the need for comfort and energy savings, which are important in continuous monitoring during ADL.

The main limitation of this work is represented by the small number of patients involved. Moreover, technical issues have prevented the use of the EMG signal, which is likely significant for FOG detection. Future research will be in the direction of increasing the available dataset and validating the present results on an extended database. Experiments will be devised in order to verify the feasibility of a minimal configuration to be tested in ADL conditions. Finally, the clinical significance of the extracted features, especially from EEG data, will be explored.

## Figures and Tables

**Table 1:** Signals used in the present study (for EEG, the International 10–20 System is employed); *IO: Electrooculogram.

Type	System	Number of Sensors	Location
28D-EEG	Wireless MOVE	28	FP1 FP2 F3 F4 C3 C4 P3 P4 O1 O2 F7 F8 P7 P8 Fz Cz Pz FC1 FC2 CP1 CP2 FC5 FC6 CP5 CP6 TP9 TP10 *IO
3D-Acc/Gyro	MPU6050	2	Lateral tibia of left leg; Wrist
1D-SC	LM324	1	Second phalanx of the index finger/middle finger of the left hand

**Table 2:** Technical characteristics of the sensors.

System	Range	Resolution	Sample Frequency
Wireless MOVE			1000 Hz
MPU6050	± 2000 dps ± 16 g	16.4 LSB/dps 2048 LSB/g	100 Hz
LM324			100 Hz

**Table 3:** Demographic and clinical information of enrolled patients (mean value  ±  standard deviation); ADL: Activity Daily Living; FOG-Q: FOG Questionnaire; UPDRS: Unified Parkinson’s Disease Rating Scale; MMSE: Mini-Mental State Examination; MOCA: Montreal Cognitive Assessment.

Subjects	12 PD
Age (years)	69 ± 7.9
Disease duration (years)	9.3 ± 6.8
ADL	81.3 ± 16.0
FOG-Q	16.2 ± 4.2
UPDRS-1	10.4 ± 5.5
UPDRS-2	16.3 ± 10.6
UPDRS-3	45.0 ± 16.0
UPDRS-4	2.2 ± 2.9
MMSE	28.2 ± 1.5
MOCA	23.6 ± 3.6

**Table 4:** Features in time and frequency domain addressed in this study; *Der1/Der2 represent the 1st and 2nd derivative signals of the SC phasic component.

Domain	Acc/Gyro Lateral Left Tibia
Frequency	Total Power, Mean Power, Max Power, STD Power, Locomotion Band Power, Freeze Band Power, Locomotion Band Power STD, Freeze Band Power STD, Freeze Index, Freeze Ratio, Skewness, Kurtosis, Energy, Entropy, Dominant Frequency, Mean Frequency, Median Frequency
Time	RMS, Mean, STD, Number of zero-crossing, Zero-crossing rate, Number of peaks, Mean distance between peaks, Mean height of the peaks, Energy, Max Amplitude, Min Amplitude, Range, Integral, Axes correlation
**Domain**	**Acc Wrist**
Frequency	*Signal magnitude*: Total Power, Mean Power, STD power, Power [0–1, 1–2, …, 15–16 Hz], Locomotion Band Power, Freeze Band Power, Power 9–12 Hz, Power 13–16 Hz *Signal components*: Total Power, Mean Power, STD Power, Max Power, Dominant Frequency, Mean Frequency, Median Frequency
Time	*Signal magnitude*: RMS, Mean, STD, Axes correlation *Signal components*: Total Power, Mean Power, STD Power, Max Power, Dominant Frequency, Mean Frequency, Median Frequency
**Domain**	**Gyro Wrist**
Frequency	*Signal magnitude*: Total Power, Mean Power, STD Power, Locomotion Band Power, Freeze Band Power *Signal components*: Total Power, Mean Power, STD Power, Max Power, Dominant Frequency, Mean Frequency, Median Frequency
Time	*Signal magnitude and components*: RMS, Mean, STD
**Domain**	**EEG**
Frequency	Total Power, Mean Power, STD Power, Skewness, Kurtosis, Energy, Entropy, Dominant Frequency, Median Frequency, Mean Frequency, Delta Band Power, Theta Band Power, Alpha Band Power, Beta1 Band Power, Beta2 Band Power, Magnitude Squared Coherence
Time	RMS, Mean, STD
**Domain**	**Phasic Component SC**
Frequency	Total Power, Mean Power, STD Power, Skewness, Kurtosis, Energy, Entropy, Dominant Frequency, Median Frequency, Mean Frequency
Time	RMS, Mean, STD, Median, Min, Max, Range, Number of local min, Number of local max
**Domain**	*Der1/Der2 Phasic Component SC
Frequency	–
Time	Mean, Median, STD, Min, Max, Range, Number of local min, Number of local max
**Domain**	**Tonic Component SC**
Frequency	Total Power, Mean Frequency, Median Frequency
Time	Slope

**Table 5:** Number of features for each signal type evaluated in this study.

Signal	# Channels	# Feature
Inertial—Lateral Left Tibia	6	186
Inertial—Wrist	6	168
EEG	18	1107
SC	1	39

**Table 6:** List of model parameters optimized in this study, along with their range of values.

Model	SVM			k-NN		
**Parameter**	kernel function	kernel scale	cost	# neighbors	distance metric	distance weight
**Value**	linear quadratic cubic gaussian	0.1–100	0.1–100	1–180	cityblock euclidean squared-euclidean	equal inverse squared-inverse

**Table 7:** Features selected by the optimized subject-independent classifier (all derived from left tibial acceleration and angular velocity sensors).

Accelerometer	r (*p*-Value)	Gyroscope	r (*p*-Value)
Kurtosis-PSD *x*-axis	−0.35 (<0.0001)	Max power *y*-axis	−0.48 (<0.0001)
Median frequency *x*-axis	0.37 (<0.0001)	Freeze ratio *y*-axis	0.46 (<0.0001)
Locomotion band power *x*-axis	−0.49 (<0.0001)	Max amplitude *y*-axis	−0.36 (<0.0001)
Freeze ratio *x*-axis	0.59 (<0.0001)	Skewness-PSD *z*-axis	−0.45 (<0.0001)
Median frequency *y*-axis	0.38 (<0.0001)	Entropy-PSD z-axis	0.58 (<0.0001)
Dominant frequency *y*-axis	0.45 (<0.0001)	Dominant frequency *z*-axis	0.40 (<0.0001)
Locomotion band power *y*-axis	−0.50 (<0.0001)	STD Locomotion band power *z*-axis	−0.50 (<0.0001)
Freeze index *y*-axis	0.37 (<0.0001)	Freeze ratio *z*-axis	0.64 (<0.0001)
Zero crossing rate *y*-axis	0.48 (<0.0001)	RMS *z*-axis	−0.55 (<0.0001)
Freeze ratio *z*-axis	0.40 (<0.0001)	P-max Max amplitude *z*-axis	−0.41 (<0.0001)
Locomotion band power *z*-axis	−0.36 (<0.0001)	Zero crossing rate *z*-axis	0.61 (<0.0001)
Zero crossing rate *z*-axis	0.35 (<0.0001)	–	–

**Table 8:** Performance of subject-independent algorithm, using uni-modal classification with accelerometer and gyroscope at the left tibial level.

(a) Lateral left tibial accelerometer.		
**Performance**	**SVM**	**kNN**
Accuracy (%)	84.11	83.40
Precision (%)	87.50	88.36
Specificity (%)	87.21	88.61
Sensitivity (%)	81.30	78.67
F-score (%)	84.26	83.23
(b) Lateral left tibial gyroscope.		
**Performance**	**SVM**	**kNN**
Accuracy (%)	84.44	85.13
Precision (%)	88.43	87.94
Specificity (%)	88.38	87.53
Sensitivity (%)	80.85	82.96
F-score (%)	84.47	85.38

**Table 9:** Performance of subject-independent algorithm with multi-modal classification using left lateral tibial accelerometer and gyroscope data.

Performance	SVM	kNN
Accuracy (%)	85.12	85.06
Precision (%)	88.72	89.37
Specificity (%)	88.55	89.40
Sensitivity (%)	82.20	81.11
F-score (%)	85.23	85.04

**Table 10:** Performance of subject-dependent algorithm with minimal and complex setup configuration for subjects 1-4-7-8-10-11.

Performance	Minimal Setup		Complex Setup	
	kNN	SVM	kNN	SVM
Accuracy (%)	84.59	85.71	87.65	88
Sensitivity (%)	82.65	81.76	86.04	85.14
Precision (%)	86.18	84.49	88.86	87.71
Specificity (%)	82.60	87.23	86.13	88.38
F-score (%)	82.63	84.41	86.08	86.73

**Table 11:** Number of true and detected episodes with minimal setup configuration.

Subject	Episodes	Length (Range) (s)	Episodes Detected with SIA	Episodes Detected with SDA
1	22	12.12 (3.3–35.4)	22	19
2	1	3.3	1	–
3	33	52.33 (3.3–238.5)	32	32
4	15	9.22 (4.5–25.20)	15	8
6	22	16.5 (5.4–32.4)	22	21
7	28	12.02 (3.3–43.5))	27	27
8	44	19.98 (3.3–58.20)	39	37
9	22	4.25 (3.3–8.4)	7	0
10	30	25.48 (4.2–64.20)	30	30
11	36	12.58 (3.3–45)	26	34
12	11	22.42 (4.5–46.5)	11	11
Tot	264	17.29 (3.79–54.6)	232	219

## Data Availability

Data available from https://data.mendeley.com/datasets/r8gmbtv7w2/3 (accessed on 1 October 2021).

## Abbreviations

The following abbreviations are used in this manuscript:

ADL	Activities of Daily Living
ECG	Electrocardiogram
EEG	Electroencephalogram
EMG	Electromiogram
EOC	Electrooculogram
FN	False Negative
FOG	Freezing Of Gait
FOG-Q	Freezing Of Gait Questionnaire
FP	False Positive
FS	Feauture Selection
kNN	k-Nearest Neighbor
LOSO	Leave-One-Subject-Out
LOTO	Leave-One-Task-Out
MDS	Movement Disorder Society
ML	Machine Learning
MMSE	Mini-Mental State Examination
MOCA	Montreal Cognitive Assessment
MSC	Magnitude Squared Coherence
MV	Majority Voting
PD	Parkinson disease
SC	Skin Conductance
SCR	Skin Conductance Response
SCL	Skin Conductance Level
SDA	Subject Dependent Algorithm
SIA	Subject Independent Algorithm
SVM	Support Vector Machine
TP	True Positive
TN	True Negative
UPDRS	Unified Parkinson’s Disease Rating Scale
